# Mycobacterium marinum as a rare cause of soft tissue infections of the upper extremity: A multidisciplinary multicenter approach to symptoms, diagnosis and treatment

**DOI:** 10.3205/iprs000197

**Published:** 2026-09-25

**Authors:** Antek Nicklas, Carsten Baade, Susann Rößler, Adrian Dragu, Tim Fülling

**Affiliations:** 1 UniversityCenter for Orthopedics, Trauma and Plastic Surgery, Faculty of Medicine and University Hospital Carl Gustav Carus, TU Dresden, Dresden, Germany; 2 Helios Weißeritztal-Kliniken GmbH, Freital, Germany; 3 Institut für Medizinische Mikrobiologie und Virologie, Universitätsklinikum Carl Gustav Carus an der TU Dresden, Dresden, Germany

**Keywords:** hand infection, hand surgery, inflammation, fish tank finger, mycobacterium marinum

## Abstract

**Background:**

Infections with *Mycobacterium* *marinum* (*M. Marinum*) are rare but clinically significant and difficult to diagnose skin and soft tissue infections, typically occurring after contact with contaminated water. The hand is most frequently affected, particularly in individuals with regular exposure to aquariums, leading to the term “fish tank finger”. This study explores the pathogenesis, epidemiology, clinical manifestations, diagnosis, and treatment of *M. Marinum* infections of the hand and presents a case series of seven patients from Eastern Saxony, including a region-specific antibiotic susceptibility analysis.

**Materials and methods:**

This retrospective multicenter study examines all seven cases of *M. Marinum* infections of the extremities between 2019 and 2024. We conducted an analysis of available antibiograms to identify regional characteristics of *M. Marinum* infections and establish a standardized treatment approach for patients in Eastern Germany.

**Results:**

In this study, seven *M. Marinum* infections were documented between 2019 and 2024. The average patient age was 65.5 years, with five male and two female patients. Infections primarily affected the hand (71.4%), with the average wound size being 2.84 cm². The mean time to diagnosis was 3.64 months. Notably, 57.1% of patients reported aquarium ownership, and two cases were linked to water exposure abroad. All patients received a three-month dual antibiotic therapy, with clarithromycin and either rifampicin or ethambutol. No recurrences were observed. Antibiotic susceptibility testing showed uniform minimal inhibitory concentration (MIC) values for rifampicin, clarithromycin, and rifabutin. Surgical debridements were performed in two cases in total.

**Conclusion:**

Upper limb infections caused by *M. Marinum* are an important differential diagnosis in cases of persistent skin lesions following water exposure. Early diagnosis and targeted therapy are essential to prevent complications. For infections in Eastern Germany, we recommend a radical surgical debridement combined with adjunctive antibiotic therapy consisting of Clarithromycin (500 mg twice daily) along with either rifampicin (600 mg daily) or rifabutin (150 mg twice daily) for a minimum duration of three months.

## Zusammenfassung

**Hintergrund:**

Infektionen mit *Mycobacterium marinum* (*M. Marinum*) sind seltene, jedoch klinisch bedeutsame und diagnostisch anspruchsvolle Haut- und Weichteilinfektionen, die typischerweise nach Kontakt mit kontaminiertem Wasser auftreten. Am häufigsten ist die Hand betroffen, insbesondere bei Personen mit regelmäßigem Kontakt zu Aquarien, weshalb der Begriff „fish tank finger“ geprägt wurde. Diese Studie untersucht die Pathogenese, Epidemiologie, klinischen Manifestationen, Diagnostik und Therapie von *M.-marinum*-Infektionen der Hand und präsentiert eine Fallserie von sieben Patienten aus Ostsachsen einschließlich einer regionsspezifischen Analyse der Antibiotikaresistenz.

**Material und Methoden:**

In dieser retrospektiven multizentrischen Studie wurden alle sieben Fälle von *M.-marinum*-Infektionen der Extremitäten zwischen 2019 und 2024 untersucht. Zudem erfolgte eine Analyse der verfügbaren Antibiogramme, um regionale Besonderheiten der *M.-marinum*-Infektionen zu identifizieren und einen standardisierten Therapieansatz für Patienten in Ostdeutschland zu etablieren.

**Ergebnisse:**

Zwischen 2019 und 2024 wurden sieben *M.-marinum*-Infektionen dokumentiert. Das durchschnittliche Patientenalter betrug 65,5 Jahre; fünf Patienten waren männlich und zwei Patientinnen weiblich. Die Infektionen betrafen überwiegend die Hand (71,4%), wobei die durchschnittliche Wundgröße 2,84 cm² betrug. Die mittlere Zeit bis zur Diagnosestellung lag bei 3,64 Monaten. Bemerkenswert ist, dass 57,1% der Patienten Aquarienbesitzer waren und zwei Fälle mit Wasserexposition im Ausland assoziiert waren. Alle Patienten erhielten eine dreimonatige duale antibiotische Therapie mit Clarithromycin in Kombination mit Rifampicin oder Ethambutol. Rezidive wurden nicht beobachtet. Die Resistenztestung zeigte einheitliche minimale Hemmkonzentrationen (MHK) für Rifampicin, Clarithromycin und Rifabutin. Insgesamt wurden in zwei Fällen chirurgische Débridements durchgeführt.

**Schlussfolgerung:**

Durch *M. Marinum* verursachte Infektionen der oberen Extremität stellen bei persistierenden Hautläsionen nach Wasserexposition eine wichtige Differenzialdiagnose dar. Eine frühzeitige Diagnosestellung und gezielte Therapie sind essenziell, um Komplikationen zu vermeiden. Für Infektionen in Ostdeutschland empfehlen wir ein radikales chirurgisches Débridement in Kombination mit einer adjuvanten antibiotischen Therapie bestehend aus Clarithromycin (500 mg zweimal täglich) sowie entweder Rifampicin (600 mg täglich) bzw. Rifabutin (150 mg zweimal täglich) über einen Zeitraum von mindestens drei Monaten.

## Introduction

Infections with *Mycobacterium marinum* (*M. Marinum*) represent a rare but clinically significant form of skin and soft tissue infections, primarily occurring after exposure to contaminated water [1]. The hand is one of the most frequently affected body regions due to its frequent contact with aquatic environments [2]. Anamnestically, contact with aquariums or fish is often identified, leading to the term “fish tank finger” in English-speaking countries [3].

This study examines the pathogenesis, epidemiology, clinical manifestations, diagnosis, and treatment of *M. Marinum* infections of the hand and presents a case series of seven patients over five years from two medical centers in Eastern Saxony, Germany. Additionally, we provide a region-specific antibiogram for *M. Marinum *infections.

*M. Marinum* is a nontuberculous mycobacterium (NTM) first isolated in 1926. It is found in both freshwater and saltwater, particularly in aquariums, swimming pools, and natural bodies of water [4]. Soft tissue infections typically occur after a subtle skin injuries that allow the bacterium to enter the body [5]. The incidence is low (~0.15 per 100,000 inhabitants/year in a Dutch cohort), and case numbers in studies are usually small [6]. In a German monocentric study by Strobel et al., only 18 cases of *M. Marinum* infection were detected in a single center in southern Germany over a period of 20 years [7]. The estimated annual incidence in the USA is 0.27 cases per 100,000 adult patients [8]. A study from Denmark by Holden et al. reported an incidence of 0.04–0.06 per 100,000 person-years between 2004 and 2009 [9]. Recently, Li et al. demonstrated that *M. Marinum* infections may also occur in outbreak settings, reporting a 32-case cluster linked to a single seafood-related exposure source, thereby highlighting the potential for common-source transmission despite the typically sporadic nature of the disease [10].

## Background

### Clinical features

*M. Marinum* is a facultative intracellular pathogen capable of replicating within macrophages. Histopathologically, *Mycobacterium marinum* infection is associated with a chronic granulomatous inflammatory response consisting of epithelioid histiocytes and occasional multinucleated Langhans giant cells, while central or caseous necrosis is variable and often absent, particularly in superficial cutaneous lesions. The bacterium prefers temperatures between 28–32°C, which explains why infections are often limited to cooler areas of the skin and extremities [11]. Clinical symptoms typically appear 2–4 weeks after exposure. In the hand, the infection manifests in a variety of ways. Characteristic changes include localized lesions that initially present as erythematous or violet papules and may develop into ulcerating nodules or plaques over time. In some cases, a sporotrichoid spread occurs, where the infection progresses along the lymphatic vessels, mimicking the characteristic lesions caused by the fungal pathogen *Sporothrix schenckii*. In immunosuppressed patients, the infection may extend to deeper tissues, including tendons, joints, and bones, leading to a significantly more severe prognosis [3]. Systemic infections, however, are often limited to immunosuppressed patients [12].

### Symptoms of M. marinum infections

Additional symptoms of hand infections generally include swelling, pain, and erythema, often accompanied by limited mobility of the affected fingers or wrists, in line with the well-known Kanavel’s signs [13]. Regarding the expected spectrum of hand infections, Hyatt and colleagues reported in a collective study that approximately one-third of cases involve Staphylococcus infections, with around 15% testing positive for MRSA and 20.2% being mixed infections. The same study noted an absence of detectable pathogens in 32.4% of cases. This category includes *M. Marinum* infections, as their detection requires specialized testing beyond standard diagnostics [14]. In our clinic, the rate of MRSA-positive cases is well below 5%.

*M. Marinum *infections often present as slowly growing, painful nodules or plaques with a chronic progression. Unlike other infections, *M. Marinum* responds poorly to standard antibiotics, necessitating targeted microbiological diagnostics and specific antimicrobial therapy. The diagnosis of hand and finger infections requires a structured approach to accurately identify the causative agent and to initiate targeted therapy. The diagnostic process is based on a thorough patient history, with particular attention to injuries, exposure to potential infection sources (in this case contaminated water or aquariums), occupational activities or hobbies. Clinical examination includes inspection and palpation of the affected areas, focusing on swelling, redness, tenderness, localized warmth, and possible purulent discharge. Imaging techniques, such as X-rays, help exclude or confirm deeper tissue involvement, such as osteomyelitis or joint effusions, and detect radiopaque foreign bodies. The most sensitive method for detecting the presence of osteomyelitis is magnetic resonance imaging (MRI) [15].

Laboratory tests, including C-reactive protein (CRP) and leukocyte count, provide insights into the level of inflammation and can support the suspected diagnosis of infection. However, CRP and leukocyte count are often not significantly elevated in mycobacterial infections, and there is no correlation between leukocyte number and CRP level in tuberculosis patients [16].

### Specific diagnostics for suspected M. marinum infection and differential diagnosis

The specific diagnosis of *M. Marinum* infections is often challenging and requires a combination of clinical suspicion, microbiological, and histopathological examinations. A key diagnostic clue is the patient’s history, particularly in those with exposure to contaminated water, fish ponds, or aquariums. The cultivation of the pathogen is performed on specialized media, such as Löwenstein-Jensen agar, incubated at 28–32°C, as these temperatures are optimal for bacterial growth [17]. However, the incubation time for samples to detect mycobacteria is 3–4 weeks. In-house, due to the prolonged incubation period, samples suspected of mycobacteriosis are incubated for a total of 8 weeks [18]. In the diagnostic workup of chronic skin and soft tissue infections, other differential diagnoses besides nontuberculous mycobacteria should be considered. These include nocardiosis, an infection caused by weakly acid-fast, branched bacteria, which often occurs after trauma and is diagnosed by culture and molecular biological methods [19], as well as *actinomycosis*, which is typically characterized by fistulous lesions and so-called sulfur granules and is confirmed histologically and by culture [20]. Other important differential diagnoses include sporotrichosis with characteristic lymphangitic spread [1], *chromoblastomycosis*, caused among other things by *Cladophialophora* species and histologically characterized by the presence of so-called Medlar bodies [21], [22], sarcoidosis as a granulomatous systemic disease with exclusion diagnosis in case of negative microbiological findings [23], and Langerhans cell histiocytosis, which is diagnosed immunohistochemically by CD1a- and Langerin-positive cells [24].

Microscopic examination using Ziehl-Neelsen staining reveals acid-fast bacilli, which are characteristic of *M. Marinum*. Molecular biological techniques, such as polymerase chain reaction (PCR), allow for rapid and specific identification of the pathogen and are particularly useful for organisms that are difficult to culture. Histological analysis often shows a granulomatous inflammatory response with detectable acid-fast bacilli, further supporting the suspicion of an *M. Marinum* infection [25].

This combination of fundamental and specialized diagnostics enables an accurate diagnosis, allowing for the initiation of targeted therapy.

### Therapy

The treatment of hand infections, including those caused by *M. Marinum*, requires a systematic and multidisciplinary approach to achieve optimal outcomes. In general hand infections, surgical management is the primary intervention. This includes thorough debridement, exploration of infected structures, irrigation of affected areas, and removal of pus and necrotic tissue. These measures are essential to reduce the infectious burden and to promote healing. In addition, an initial empirical antibiotic therapy is started, targeting the most common pathogens of hand infections, and is later adjusted based on pathogen identification. For intravenous antibiotic administration, ampicillin-sulbactam (2,000 mg/1,000 mg) three times daily is recommended. Alternatively, oral amoxicillin/clavulanic acid (875 mg/125 mg) can be administered three to four times daily for at least five days. Our therapeutic algorithm was implemented in accordance with the recommendations of the Paul-Ehrlich-Institut and our internal hospital standard operating procedure (SOP) [26]. 

For *M. Marinum* infections, treatment is more specialized and often requires a combination of pharmacological therapy and surgical intervention [27], [28]. Published evidence indicates that debridement alone may be inadequate for managing infections with lymphatic spread in suggested *M. Marinum* infections [27]. The recommended antibiotic therapy consists of a dual-drug regimen with clarithromycin and RMP for several months, tailored to wound depth and healing progress. Literature suggests a prolonged treatment duration of at least 3–4 months [8]. While isoniazid resistance rates of 8–12% have been described for *Mycobacterium tuberculosis*, *Mycobacterium marinum* is intrinsically resistant to isoniazid. In vitro studies show isoniazid MIC50 values of 4 µg/ml and MIC90 values of 8 µg/ml (above therapeutic concentrations) [29].The underlying mechanism lies in the inefficient activation of the drug by the enzyme KatG, which is markedly reduced in *M. Marinum* compared to *M. tuberculosis* [30].

The combination may be supplemented with EMB to enhance efficacy through synergistic effects [31]. In a large cohort of 200 clinical isolates, high susceptibility was observed for clarithromycin, rifampicin, rifabutin, moxifloxacin, linezolid and trimethoprim-sulfamethoxazole (82.5–100%), whereas susceptibility to tetracyclines and ciprofloxacin was moderate [32]. In vitro antimicrobial susceptibility testing of *M. Marinum* demonstrates a characteristic pattern with consistent low MIC values for rifampicin, rifabutin and clarithromycin, while several other agents, including fluoroquinolones and tetracyclines, show variable activity [33].

For deep skin and soft tissue infections caused by *M. Marinum*, a combined approach of pathogen-directed antimicrobial therapy and surgical management is commonly employed, particularly in cases with extensive tissue involvement. Surgical intervention is generally reserved for complicated courses, such as abscess formation, involvement of deeper structures (e.g. tendon sheaths, joints or bone), or failure of conservative therapy. In these situations, surgical debridement or excision of affected tissue may be necessary to reduce the bacterial load and to support effective antimicrobial treatment. However, standardized, evidence-based recommendations regarding the timing and extent of surgical intervention are lacking, and treatment decisions are usually based on disease severity, anatomical involvement, and clinical response.

Therapeutic monitoring is conducted through regular clinical follow-ups and imaging techniques to assess the infection’s progression and treatment effectiveness. This integrative approach ensures that both acute symptoms and potential long-term complications are effectively managed.

## Material and methods

This retrospective cohort study included patients with soft tissue infections of the upper extremity caused by *Mycobacterium marinum* who were treated in participating centers. Inclusion criteria were:

- Definitive microbiological confirmation of *M. Marinum* by polymerase chain reaction (PCR) with subsequent validation by the reference laboratory in Borstel, and
- Age greater than 18 years at the time of follow-up.

Patients were excluded if they were younger than 18 years, if *M. Marinum* could not be detected, if another pathogen was identified, or if microbiological confirmation of *M. Marinum* was unavailable. Only PCR-confirmed cases were considered; suspected cases without microbiological confirmation were also excluded.

Clinical characteristics, diagnostic findings, microbiological results, and therapeutic regimens were retrospectively analyzed. The retrospective evaluation was conducted in accordance with the Declaration of Helsinki. Ethical approval for the study was obtained from the local ethics committee of TU Dresden (BO-ff (Mono)-EK-217052025). All patients provided informed consent to participate in the study. Written informed consent for the use of clinical data and photographic documentation for research purposes was obtained from all patients included in the study. The clinical data were retrospectively extracted from the electronic hospital information system Orbis (Agfa HealthCare, Mortsel, Belgium). Patients with suspected or confirmed infections caused by *M. Marinum* were identified through a targeted search within the hospital information system from 2019–2024. The patient records thus identified were subsequently subjected to a peer-review process, with two independent reviewers manually verifying and evaluating the data to ensure completeness and accuracy. Additionally, an in-hospital search was carried out within our microbiological database to identify *M. Marinum* infections affecting the extremities. For this purpose, microbiological reports and antimicrobial susceptibility testing results provided by the National Reference Center for Mycobacteria in Borstel (Germany) were reviewed.

For the determination of the minimum inhibitory concentrations (MICs) of *M. Marinum*, the broth microdilution method was performed at the National Reference Center for Mycobacteria in Borstel according to the guidelines of the Clinical and Laboratory Standards Institute (CLSI M24). Antimicrobial susceptibility testing results were extracted from the laboratory reports and analyzed descriptively. In two cases, where the disease was presumably acquired outside of Germany, no antibiogram was available.

As only adult participants (over 18 years of age) were involved in the study, obtaining informed consent from parents or legally authorized representatives was not required.

## Results

### Case series

In this study, we present a case series of seven *M. Marinum* infections documented between 2019 and 2024. The average age of the patients was 65.5 years (±14.1 years), with five male and two female patients. In four cases, the right side was affected (Figure 1 (Fig. 1)). The hand was involved in five cases, while one case affected the upper arm and another the forearm. The average wound size was 2.84 cm² (±1.35 cm²), measured at initial and defined as the central lesion (skin defect with porus), explicitly excluding the surrounding erythema as a reactive perilesional response rather than part of the wound itself. The mean time from the first medical contact to diagnosis was 3.64 months (±3.73 months). Notably, 57.1% of patients retrospectively reported owning an aquarium. In two cases, a recent stay abroad with water exposure was suspected as the source of infection, while in one case, the source remained unclear despite a thorough medical history. Of the seven included patients, two underwent surgical intervention. Surgery was performed only in these cases due to the prolonged interval between infection onset and pathogen identification. Two patients presented with joint infections, while bone involvement or osteolysis was not observed. The mean duration of surgery was 73±14 minutes. The remaining four patients were managed non-operatively, as they had soft tissue infections without abscess formation.

Regarding antibiotic therapy, all patients received dual antibiotic treatment. Clarithromycin was administered to all patients. In two cases of renal insufficiency, the dose was reduced by 50% from 500 mg twice daily mg to 250 mg twice daily. As the second antibiotic, three patients received ethambutol (EMB) (400 mg once daily), three patients received rifampicin (600 mg once daily), and one patient received rifabutin (300 mg daily; 150 mg twice daily) due to its lower potential for CYP-mediated drug interactions. The duration of antibiotic therapy was three months in all cases, and no recurrences were observed. Patient data are provided in Table 1 (Tab. 1).

Moreover, the pathogens exhibited uniform susceptibility profiles and minimum inhibitory concentrations across all tested samples.

Rifampicin, which was administered in four cases, showed an average minimum inhibitory concentration (MIC) of 0.294 µg/mL (Range 0,12–0,5 µg/mL). Similarly, clarithromycin exhibited an MIC of 0.875 µg/mL (Range 0,5–1 µg/mL). For rifabutin, the mean MIC was 0.25 µg/mL across all samples.

The remaining minimum inhibitory concentrations and patient data are presented in Table 2 (Tab. 2).

## Discussion

The prognosis for *Mycobacterium marinum* infections is generally favorable, provided that early diagnosis is made and appropriate, targeted therapy is initiated. However, delays in diagnosis or initially inadequate antibiotic treatment can lead to chronic courses with progressive tissue destruction, persistent infections, and permanent functional limitations of the affected hand or fingers. Particularly when deep structures such as tendon sheaths, joints, or bones are involved, protracted courses have been described that require repeated surgical interventions and prolonged antimicrobial therapy [3], [25]. Preventive measures therefore play a crucial role in avoiding *M. Marinum* infections. These include, in particular, avoiding skin injuries when working with potentially contaminated water and wearing protective gloves when handling aquariums or fish, which should be considered standard practice. Furthermore, early cleaning and disinfection of even the smallest skin lesions are essential to prevent the pathogen from entering the body and to minimize the risk of subsequent infections. Regarding the spectrum of bacterial pathogens, the cases examined from eastern Saxony showed no significant outliers in the resistance patterns of *M. Marinum* isolates. The antimicrobial susceptibility pattern observed in our cohort is in line with published data from Aubry et al., based on clinical isolates from France, as well as with the review by Wang et al. from China, both consistently demonstrating strong activity of rifamycins and macrolides against *M. Marinum* [33], [34]. In this multicenter case series, the antimicrobial susceptibility patterns of seven *M. Marinum* isolates from clinically relevant hand infections were analyzed. Despite the limited number of cases, the evaluation of individual minimum inhibitory concentrations (MICs) allows for a differentiated assessment of regional susceptibility and its clinical relevance. The lowest and most consistent MIC values were found for rifampicin (0.12–0.5 µg/mL) and rifabutin (0.25 µg/mL), highlighting the high in vitro activity of rifamycins against *M. Marinum*. These results are consistent with previous in vitro studies and clinical case series that describe rifamycins as key components of therapy, particularly in deep or complicated infections [29], [35]. Clarithromycin also showed favorable MIC values between 0.5 and 1 µg/mL, thus confirming its role as a mainstay of combination therapy in cutaneous *M. Marinum* infections [36], [37]. 

In contrast, more than 50% of the isolates showed elevated MIC values for doxycycline (2–4 µg/mL), suggesting limited efficacy of tetracycline-based therapies in this range. Similar variability was also observed for fluoroquinolones, particularly ciprofloxacin (2–4 µg/mL), confirming previous reports of inconsistent sensitivity in this class of drugs [35]. Cotrimoxazole showed overall low in vitro activity with MIC values of 10–19 µg/mL and therefore appears to be of limited suitability as a therapeutic option [29]. It was striking, however, that elevated MIC values for doxycycline were detectable in more than 50% of the isolates examined, compared to international standard values. Whether this represents a regionally specific resistance pattern cannot be definitively determined based on the limited number of cases, but it should be considered when choosing an antibiotic in eastern Germany. 

## Conclusion

*Mycobacterium marinum* hand infections represent a rare but clinically relevant differential diagnosis in patients presenting with chronic, non-healing skin lesions following water exposure. Early diagnosis and the initiation of targeted antimicrobial therapy are essential to prevent chronic infection, progressive tissue destruction, and long-term functional impairment of the affected hand or fingers. Our data suggest a representative pattern of regional antibiotic susceptibility in Saxony and Eastern Germany, as the observed minimum inhibitory concentrations were largely consistent across cases. Notably, more than 50% of the isolates demonstrated elevated MIC values for doxycycline, indicating a potentially reduced efficacy of tetracycline-based regimens in this region.

Our retrospective analysis further suggests that combination therapy with clarithromycin and a second antibiotic, such as rifabutin or rifampicin, appears to be an effective adjunctive treatment following surgical debridement in cases with deeper tissue involvement or complicated disease courses. Surgical exploration and debridement should be considered particularly in patients with joint involvement, tenosynovitis and deep abscess formation. In contrast, superficial cutaneous lesions without evidence of deeper extension may often be managed with antimicrobial therapy alone. This multimodal approach is in line with published clinical series reporting favorable treatment outcomes for *M. Marinum* infections when managed with appropriate antimicrobial regimens, often in combination with surgical intervention. Reported cure rates range from approximately 75% to 90%, while relapses are uncommon and long-term functional impairments are rarely observed [6], [9], [38], [39]. Given the increasing popularity of aquariums and water-based recreational activities, heightened clinical awareness and public education regarding this rare but potentially severe infection are essential. Increased vigilance among healthcare professionals and individuals at risk may facilitate earlier diagnosis, timely initiation of appropriate therapy, and ultimately improved patient outcomes with preservation of hand function. 

### Limitation

First, the small sample size limits the generalizability of the findings. Second, the retrospective design introduces potential selection and information bias. Third, functional outcome data were not consistently available, and standardized assessment of functional results was lacking. Additionally, clear criteria for defining definitive clinical healing were not uniformly applied. Finally, two antibiograms were missing, which may have affected the accuracy of microbiological interpretation and treatment evaluation.

## Notes

### Ethical approval

This retrospective multicenter study was conducted in accordance with the principles of the Declaration of Helsinki. Ethical approval was obtained from the local ethics committee of TU Dresden (BO-ff (Mono)-EK-217052025).

### Informed consent

Written informed consent for the use of clinical data and photographic documentation for research purposes was obtained from all patients included in this study.

Written informed consent for the publication of anonymized clinical data and images was obtained from all patients included in this study.

### Author contributions

AN/TF conceived the study, participated in the study design, performed the statistical analysis and drafted the manuscript. AD & CB contributed to data collection and the statistical interpretation. AN, TF, SR, AD, CB participated in the study design and oversaw the manuscript drafting process. All authors read and approved the final manuscript.

### Competing interests

The authors declare that they have no competing interests.

### Availability of data and materials

The datasets generated and analyzed during the current study are available from the corresponding author on reasonable request.

## Figures and Tables

**Table 1:**
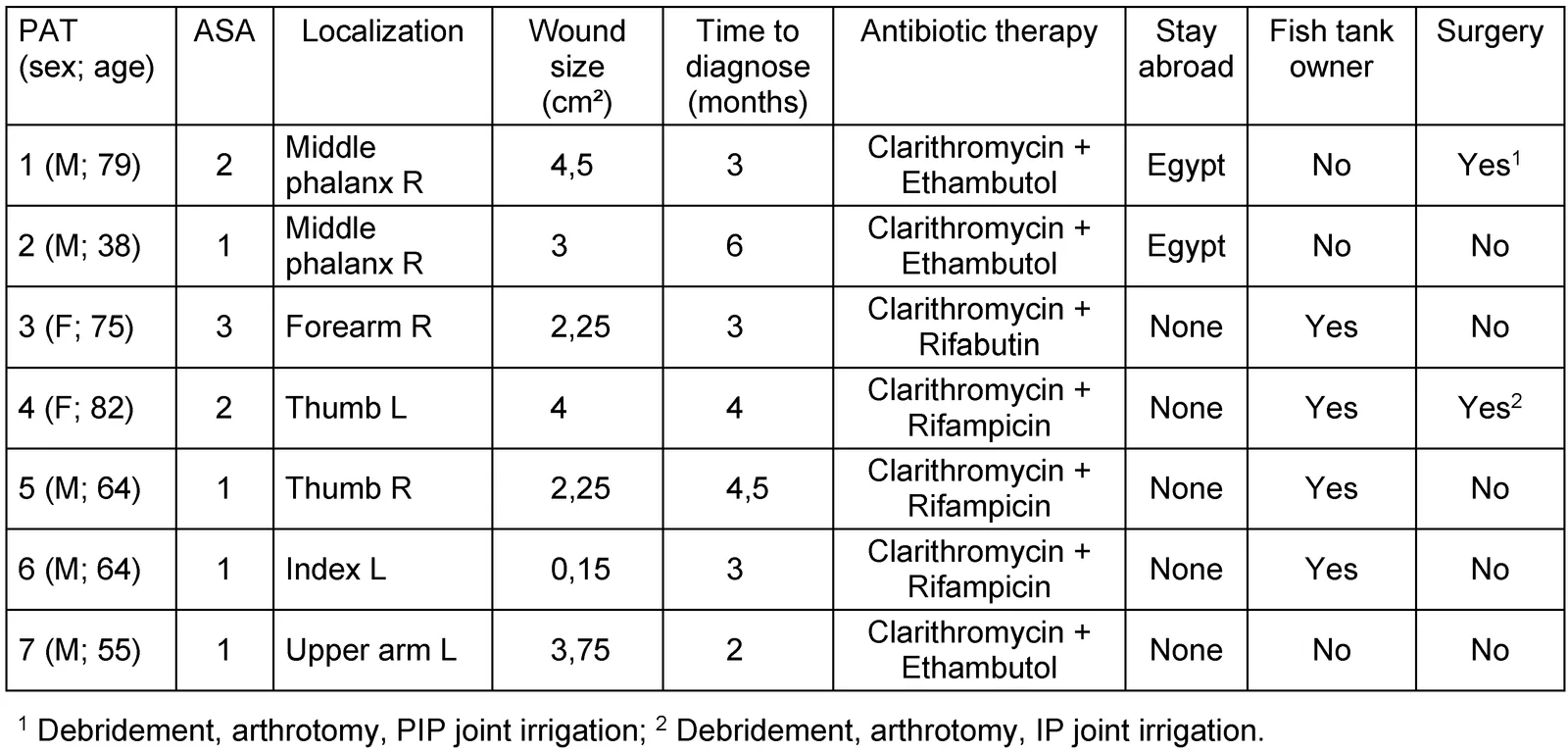
Patient characteristics. All patients received a standardized antibiotic regimen for a total duration of three months. Clarithromycin was administered at a dosage of 500 mg twice daily, ethambutol at 400 mg once daily, rifampicin at 600 mg once daily, and rifabutin either at 300 mg once daily or 150 mg twice daily.

**Table 2:**
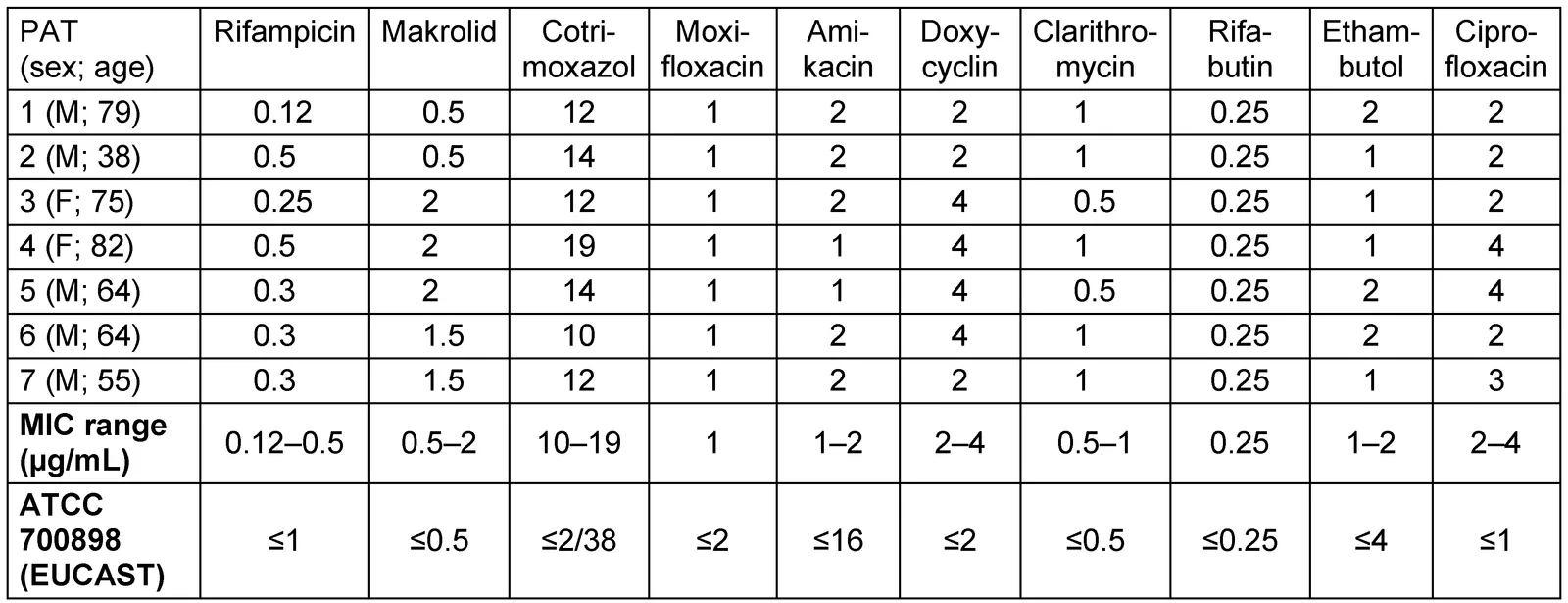
Antimicrobial susceptibility testing results for *Mycobacterium marinum* isolates from the study cohort. Minimum inhibitory concentrations (MICs) are reported in µg/mL. Susceptibility testing was performed at the National Reference Center for Mycobacteria (Borstel, Germany) using the broth microdilution method according to CLSI M24 guidelines. The ATCC 700898 strain was used as quality control according to EUCAST recommendations.

**Figure 1:**
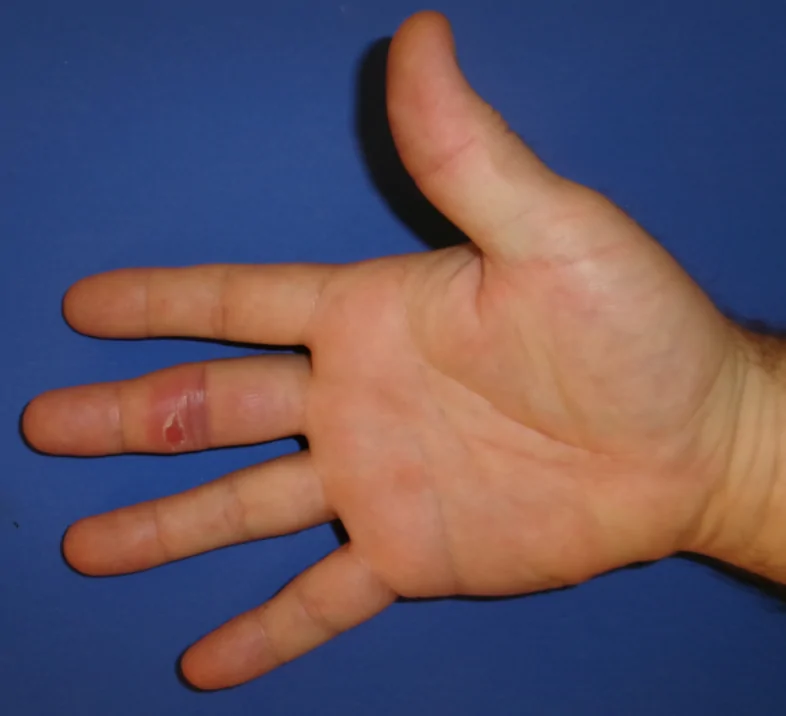
*M. Marinum* infection of the PIP-Joint of the middle finger
